# Technique of bilateral internal thoracic artery minimally invasive coronary artery bypass grafting with double-lung ventilation

**DOI:** 10.1016/j.xjtc.2023.05.008

**Published:** 2023-05-26

**Authors:** Anna Kathrin Assmann, Stephan Urs Sixt, Artur Lichtenberg, Alexander Assmann

**Affiliations:** a Department of Cardiac Surgery, Heinrich Heine University, Duesseldorf, Germany; b Department of Anaesthesiology, Heinrich Heine University, Duesseldorf, Germany

**Figure:**
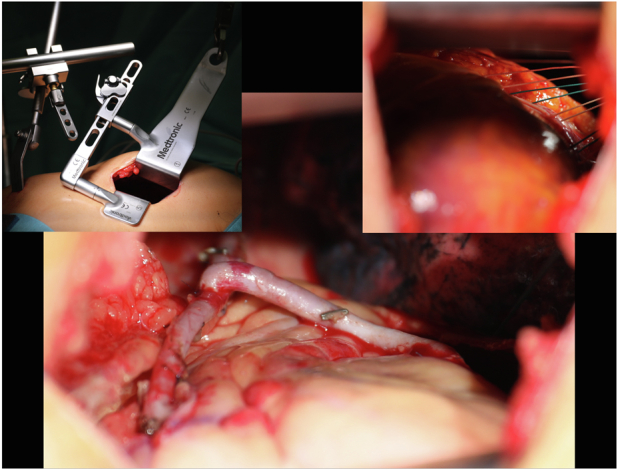
Bilateral internal thoracic arteries off-pump MICS-CABG with double-lung ventilation. © Georg Thieme Verlag KG.

**Central Message**

Anaortic off-pump MICS-CABG allows for minimally invasive revascularization with both internal thoracic arteries by means of double-lung ventilation even in patients with impaired lung function.

Minimally invasive coronary artery bypass grafting (MICS-CABG) is as safe as off-pump CABG (OPCAB) via sternotomy while allowing for superior cosmetics, wound healing, and recovery.^1^^,^^2^

MICS-CABG with conventional single-lung ventilation is limited by a variety of chronic lung diseases as well as technique-inherent pathomechanisms such as missing left lung ventilation that results in intrapulmonary shunting and consecutive hypoxemia, left lung hypoxic pulmonary vasoconstriction leading to pulmonary hypertension, and right lung enhanced airway pressure promoting acute lung injury.

Recently, we demonstrated that patients with severely impaired lung function are eligible for MICS-CABG when using a pulmonary fan.^3^ Here, we present in detail our bilateral internal thoracic artery (BITA)-MICS-CABG technique with double-lung ventilation.

## Methods

### Patient Selection

BITA-MICS-CABG requires thorough planning considering several contraindications (Table 1). A computed tomography chest scan reveals the patients' intrathoracic anatomy and thus eligibility. Institutional review board approval was not required. An informed written consent for publication of study data was obtained from the patient in the video.

**Table 1:** Contraindications for bilateral internal thoracic artery minimally invasive cardiac surgery coronary artery bypass grafting

Emergency operation
Hemodynamic instability
Severely impaired ventricular function/dilated ventricles
Cardiac redo operation
Severely stenotic or occluded subclavian artery
Severe obesity (relative contraindication)
Chest deformity (relative contraindication)

### Anesthesia and Monitoring

An anesthesiologist team with profound experience in MICS and OPCAB is mandatory. We routinely use pulmonary artery catheters to continuously monitor pulmonary arterial, central venous, and left ventricular filling pressure and cardiac output. Adequate volume management and differentiated catecholamine use are of utmost importance.

### Positioning and Incision

Patients are placed in the supine position with the left thorax lifted up by 30°. After a submammary skin incision (5-8 cm along the fifth intercostal space) and extrathoracic preparation, the parietal pleura is widely opened to reduce the risk of costal fractures (Video 1).

### Pulmonary Fan for Double-Lung Ventilation

Double-lung ventilation is achieved by our pulmonary fan technique as previously described.^3^ In brief, the mediastinal pleura is incised 1 to 2 cm anterior of the phrenic nerve, and 6 to 12 sutures are stitched along the pericardio-pleural margin and pulled laterally through the third or fourth intercostal space. Thus, the constructed fan retracts the left lung to enable continuous double-lung ventilation (Video 1).

### Graft Harvesting

The left ITA (LITA) is skeletonized. For right ITA (RITA) preparation, an additional subxiphoidal retractor lifts the sternum (Video 1).

### Anastomoses

After LITA-RITA T-graft construction, the LITA is typically sutured to the left anterior descending artery and the RITA to coronaries of the (postero) lateral wall (Video 2). For cardiac positioning and target vessel exposure, suctioning positioner devices and stabilizers are utilized. Afterward, a transit-time-flow measurement confirms adequate blood flow to the coronary targets.

## Results

**Figure E1:**
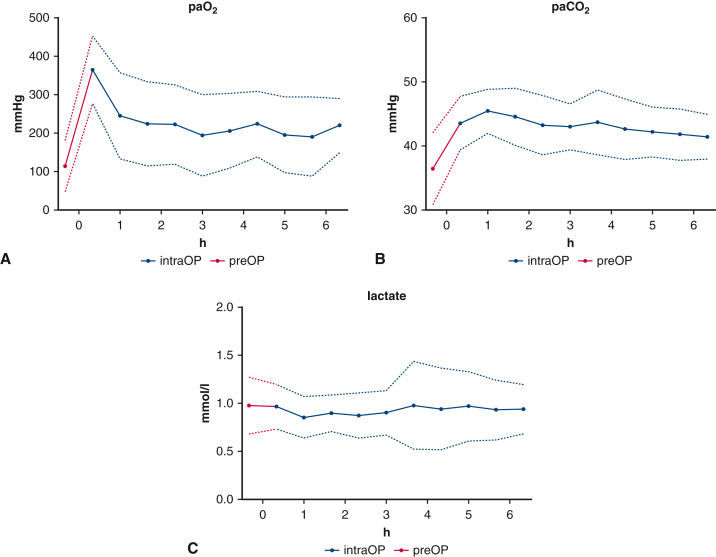
A, Pre- and intraoperative course of Pao_2_. B, Paco_2_. C, Arterial lactate levels. *intraOP*, Intraoperative; *preOP*, preoperative.

So far, 16 patients have undergone BITA-MICS-CABG in our department (baseline characteristics are presented in Table E1). All patients received a LITA-to- left anterior descending artery bypass and an RITA T-graft to either a diagonal (n = 11) or an obtuse marginal branch (n = 5). During the whole operation (cut-suture time 347.3 ± 59.6 minutes), patients showed adequate pulmonary gas exchange and aerobic metabolism (Figure E1). All patients were extubated on the day of surgery and showed regular postoperative cardiac enzymes and in-hospital outcome (Table E2).

## Discussion

BITA-MICS-CABG allows for totally arterial revascularization without aortic manipulation via anterolateral minithoracotomy. Anaortic coronary surgery decreases the risk of intraoperative stroke,^4^ and the long-term patency of arterial grafts should be considered superior to venous bypasses.^5^ Compared with OPCAB via sternotomy, MICS-CABG results in fewer wound infections, more rapid recovery, and reduced length of hospital stay.^1^

Our pulmonary fan technique guarantees continuous double-lung ventilation and adequate gas exchange without impairing the surgeon's view and working space. Thereby, even patients with impaired lung function can benefit from MICS-CABG. Furthermore, inherent issues of single-lung ventilation (ie, hypoxemia, pulmonary hypertension, and acute lung injury) may be avoided. Left lung ventilation is all the more important for BITA-MICS-CABG because right lung extension frequently has to be partially restricted during RITA preparation. Moreover, compared with minimally invasive direct CABG with only 1 anastomosis, multivessel MICS-CABG requires substantially more time so that adverse cascades triggered by single-lung ventilation have greater influence on the patient. Thus, continuous double-lung ventilation has the potential to avoid conversions to cardiopulmonary bypass and sternotomy. Actually, the usefulness of left lung ventilation to make sternal sparing coronary surgery accessible to a larger group of coronary artery disease patients has been previously shown in the context of robotic totally endoscopic bypass grafting.^6^

MICS-CABG is a complex microsurgical procedure, particularly when combined with BITA use. To guarantee a safe procedure and optimal long-term graft patency, an expert team is required, including trained cardioanesthetists. The specialization process of a MICS-CABG team should start from profound expertise in OPCAB, including complications management. Further learning steps comprise basic elements of MICS, adoption of minimally invasive direct CABG with a single anastomosis, evolution toward multivessel MICS, and finally integration of BITA preparation.

## Conclusions

BITA-MICS-CABG is an excellent, innovative approach that combines the advantages of off-pump surgery without aortic manipulation, totally arterial revascularization, and minimal invasiveness. Thus, multivessel arterial bypass grafting is realized in conjunction with reduction of surgical trauma and operative risk. The presented double-lung ventilation technique counteracts pathomechanisms inherent to MICS under single-lung ventilation and expands the spectrum of eligible patients toward those with concomitant lung diseases.

## Figures and Tables

**Table E1:** Patients' baseline characteristics (N = 16)

Variable	Result
Age (y)	64.9 ± 8.47
Sex male	16
BMI (kg/m^2^)	28.1 ± 4.79
LVEF (%)	56.3 ± 3.34
CCS	Class I: 5 Class II: 8 Class III: 3
NYHA	Class I: 7 Class II: 7 Class III: 2
AF	2
Stroke	0
CAD	5
PVD	0
COPD	6
Asthma	3
DM II	8
Creatinine (mg/dL)	1.04 ± 0.24

Values are presented as mean ± SD or n. *BMI*, Body mass index; *LVEF*, left ventricular ejection fraction; *CCS*, Canadian Cardiovascular Society classification; *NYHA*, New York Heart Association; *AF*, atrial fibrillation; *CAD*, carotid artery disease; *PVD*, peripheral vascular disease; *COPD*, chronic obstructive pulmonary disease; *DM II*, diabetes mellitus type II.

**Table E2:** Patient outcomes (N = 16)

Variable	Result
Stroke	0
CPR	0
CK max (U/L)	1128.7 ± 587.4
CK-MB max (U/L)	27.8 ± 11.6
Troponin-Ths max (ng/L)	427.6 ± 501.6
LVEF (%)	55.4 ± 3.4
Creatinine (mg/dL)	1.07 ± 0.28
CVVHDF	0
Redo thoracotomy	1∗
Redo intubation	0
30-d mortality	0

Values are presented as mean ± SD or n. *CPR*, Cardiopulmonary resuscitation; *CK*, creatine kinase; *CK-MB*, creatine kinase–cardiac isoenzyme; *Ths*, T high sensitive; *LVEF*, left ventricular ejection fraction; *CVVHDF*, chronic veno-venous hemodiafiltration.

∗ postoperative bleeding from severe pleural adhesions prepared during MICS-CABG.

## Supplementary Data

Video 1 Bilateral internal thoracic artery minimally invasive cardiac surgery coronary artery bypass grafting: Minimally invasive thoracic access, pulmonary fan technique for double-lung ventilation, and bilateral internal thoracic arteries preparation. Video available at: https://www.jtcvs.org/article/S2666-2507(23)00182-7/fulltext.

Video 2 Bilateral internal thoracic artery minimally invasive cardiac surgery coronary artery bypass grafting: Central und peripheral anastomoses generation. Video available at: https://www.jtcvs.org/article/S2666-2507(23)00182-7/fulltext.
