# Supplementary figures and images for: Technique of bilateral internal thoracic artery minimally invasive coronary artery bypass grafting with double-lung ventilation
Source: JTCVS Tech. 2023 May 26;20:87–91. doi: 10.1016/j.xjtc.2023.05.008 (PMC10405255; doi:10.1016/j.xjtc.2023.05.008)

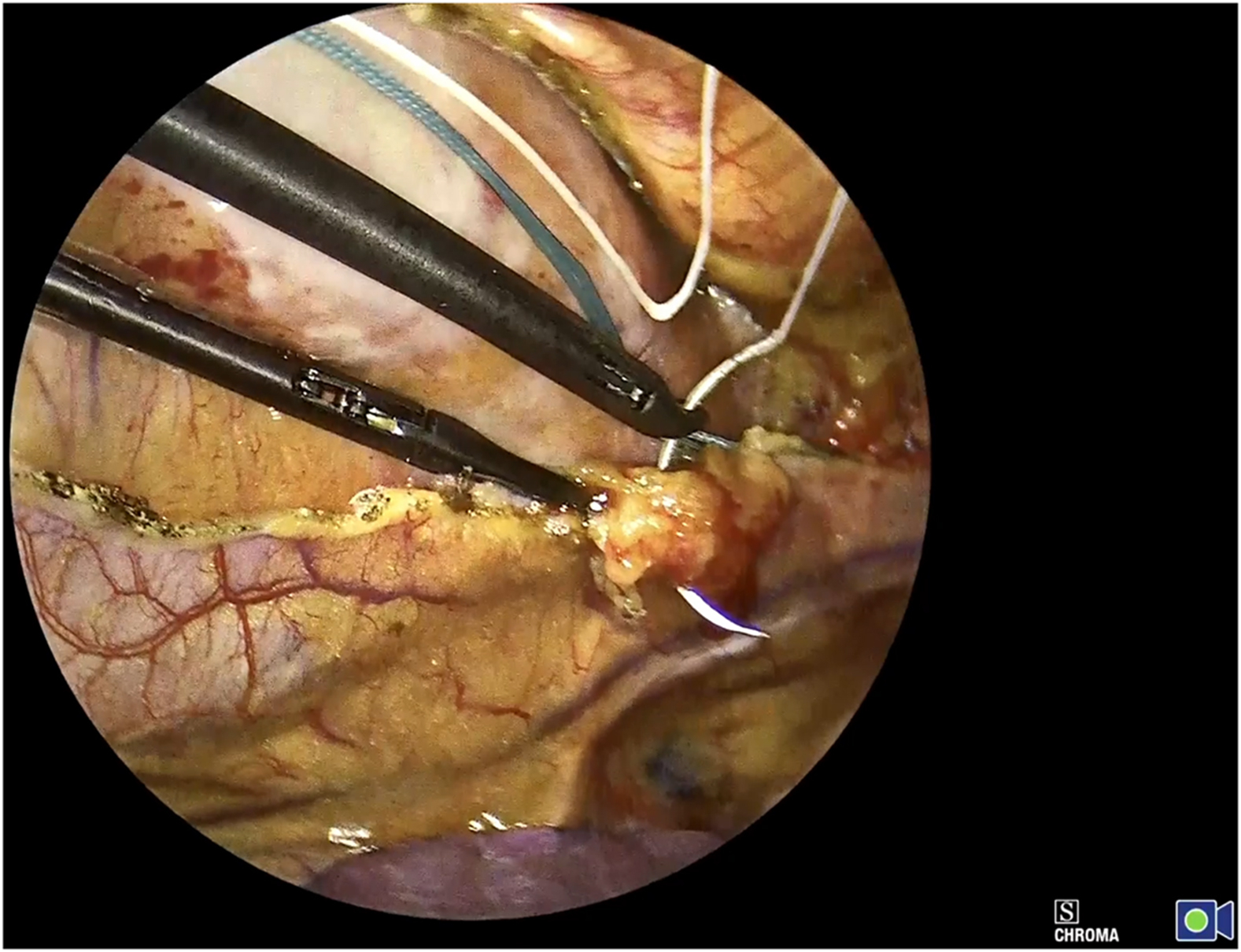

Supplement: Video 1 — Bilateral internal thoracic artery minimally invasive cardiac surgery coronary artery bypass grafting: Minimally invasive thoracic access, pulmonary fan technique for double-lung ventilation, and bilateral internal thoracic arteries preparation. Video available at: https://www.jtcvs.org/article/S2666-2507(23)00182-7/fulltext. [file fx2.jpg]

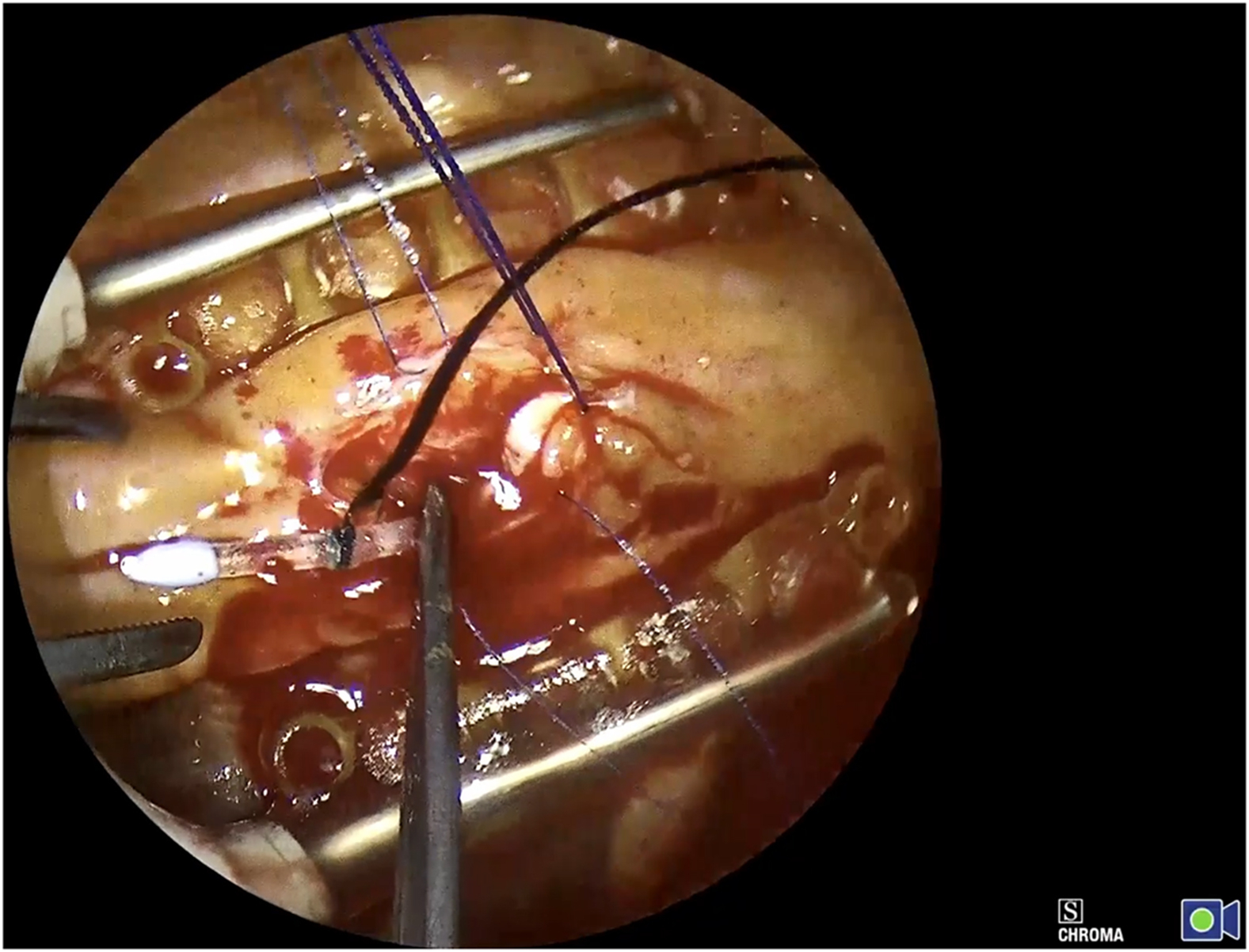

Supplement: Video 2 — Bilateral internal thoracic artery minimally invasive cardiac surgery coronary artery bypass grafting: Central und peripheral anastomoses generation. Video available at: https://www.jtcvs.org/article/S2666-2507(23)00182-7/fulltext. [file fx3.jpg]
